# Temperament, Beliefs About Pain Control, and Pain Intensity in Endometriosis Patients

**DOI:** 10.1007/s10880-016-9473-8

**Published:** 2016-10-13

**Authors:** Joanna Bylinka, Włodzimierz Oniszczenko

**Affiliations:** Faculty of Psychology, University of Warsaw, Stawki 5/7, 00-183 Warsaw, Poland

**Keywords:** Temperament, Pain control, Pain intensity, Endometriosis

## Abstract

This correlational study investigated the relationships between temperament, beliefs about pain control, and pain intensity ratings in a group of 103 women diagnosed with endometriosis. Temperament traits were assessed using the Formal Characteristics of Behaviour-Temperament Inventory. Beliefs about pain control were measured using the Polish version of the Beliefs about Pain Control Questionnaire. The Numerical Rating Scale (NRS-11) was used to measure pain intensity. There was a high negative correlation between the temperament trait of endurance and pain intensity ratings. Moderate negative correlations with pain intensity were found for internal beliefs about pain control. Hierarchical multiple regression analysis indicated that the endurance trait and internal beliefs about pain control accounted for 33 % of the variance in pain intensity ratings in women with endometriosis.

## Endometriosis and Pain

Endometriosis is the presence of the lining of the uterus (endometrium) outside the uterine cavity, and occurs most commonly in women of childbearing age. Unusual and varied symptoms of endometriosis suggest the presence of a pseudomalignant tumour, in which the basic and most severe symptom is chronic pelvic pain. The intensity and persistence of pain in endometriosis are comparable with pain in bone metastases (Fraser, 2010). Chronic pain is often associated with a number of psychological consequences, such as depression, anxiety, and sexual dysfunction (Bruckenthal, 2011). From a psychological view, the pain, aside from the components of biological agents, involves various cognitive, affective, and behavioral factors causing unpleasant emotional as well as sensory experiences [American Psychological Association (APA), 2007]. We investigated only the quantitative aspect of pain (i.e., pain intensity rating) and not its aspect of unpleasantness.

## Personality and Management of Pain

The issue of personality and beliefs about the control of chronic pain in women with endometriosis has not been widely considered in the literature. Previous studies demonstrated that women with endometriosis have a higher level of psychoticism, anxiety and introversion compared with women who suffer pain caused by other factors (Low, Edelmann, & Sutton, 1993). The presence of depressive symptoms and high levels of trait and state anxiety correlated positively with the severity of pain found in women with endometriosis (Sepulcri & Amaral, 2009). An association between posttraumatic stress disorder symptoms and the risk of endometriosis also has been demonstrated (Seng, Clark, McCarthy, & Ronis, 2006).

## Regulative Theory of Temperament Traits

The objective of our study was to investigate the association of temperament, beliefs about pain control and the assessment of the severity of pain in women diagnosed with endometriosis. We based our study on the regulative theory of temperament (RTT) formulated by Strelau (1998). This theory emphasizes the role of temperament in the regulation of people’s relations with their environment. According to the RTT, temperament refers to basic, stable personality traits. These traits are manifested in the energetic characteristics (response intensity) and temporal characteristics (speed, tempo, and mobility) of behavior. The RTT postulates six temperament traits (Strelau, 1996) as follows.

- Briskness (BR), the tendency to respond swiftly, to maintain a high tempo of activity, to switch easily from one behavior to another and to adequately respond to changes in the environment.
- Perseveration (PE), the tendency to maintain and repeat behaviors after the situation that evoked these behaviors has changed.
- Sensory sensitivity (SS), the capacity to respond to sensory stimuli whose stimulating value is low.
- Emotional reactivity (ER), the tendency to respond intensely to emotogenic stimuli, which manifests in high emotional sensitivity and low emotional resistance—that is, a low ability to control emotional reactions in response to strong emotogenic stimuli.
- Endurance (EN), the capacity to respond adequately in situations that require prolonged or highly stimulating activity and/or in conditions of high external stimulation.
- Activity (AC), the tendency to engage in behaviors that are highly stimulating or that provide highly stimulating environmental input.

## Health-Related RTT Traits

According to the RTT, a specific temperament trait or constellation (a temperamental risk factor) increases the risk of pathology, either itself, or via interaction with intensive, prolonged or frequent environmental factors (Strelau, 2008). The biological mechanisms underlying these traits interact to regulate the individual level of arousal regarding duration and intensity. This may lead to various consequences, such as behaviour disorders or psychological and somatic ill health (Strelau, 1998). The RTT traits can play a similar role in disorders as better-known personality traits, such as neuroticism and extraversion (Kandler, Held, Kroll, Bergeler, Riemann, & Angleitner, 2012), harm avoidance and reward dependence (Hornowska, 2011) or anxiety, with which they are correlated (Strelau, 2008). Emotional reactivity and perseveration (ER and PE) are conducive to intense emotional response and prolonged emotional arousal. Briskness, endurance, and activity help an individual adapt to environmental conditions. These last three traits (BR, EN, and AC) are also protective resources; they help to protect individuals from the risk of disorders, while ER and PE have been shown to be positively correlated with anxiety disorder (Oniszczenko & Laskowska, 2014; Rzeszutek, Oniszczenko, & Firląg-Burkacka, 2012; Zawadzki & Popiel, 2012). In a recent study, Fruehstorfer, Veronie, Cremeans-Smith, and Newberry (2012) expected that if high-PE individuals relived stressful events, the individuals would have higher instances of illness. The authors showed that PE can predict the occurrence of psychometrically measured somatic anxiety. Fruehstorfer et al., using an illness checklist, also showed that interactions between PE and other traits (PE × SS; PE × ER; PE × EN) can predict the occurrence of diagnosable illnesses that are nongender specific (common illnesses, respiratory illnesses, inflammations, injury, and urinary tract infections).

ER and PE are positively correlated with distress, as measured by Veit and Ware’s Mental Health Inventory (MHI) but negatively with MHI psychological well-being (Strelau, 2008). Studies have also showed that BR, EN and AC are positively correlated with MHI psychological well-being, while BR, EN and AC are negatively correlated with anxiety disorders and MHI distress. Thus far, it has been demonstrated that ER is negatively associated with positive mood level (Jankowski & Zajenkowski, 2012).

A recent meta-analytic review showed that individuals living with chronic pain experience significant problems with depression and anxiety, which increases their fear of pain (Burke, Mathias, & Denson, 2015). Pain symptoms do not always correlate with the severity of endometriosis, suggesting that emotional distress and other psychological factors may play a role in pain perception in women with endometriosis (Szendei, Hernádi, Dévényi, & Csapó, 2005; Vercellini et al., 2007). However, Markovic, Manderson, and Warren (2008) emphasized that women’s ability to endure pain and endure treatment procedures throughout the entire process of diagnosis and treatment of endometriosis is important for their well-being. Therefore, we expect that ER, PE, and EN, as RTT traits, and to a lesser extent BR and AC, may be related to pain intensity ratings in women with endometriosis. It has been demonstrated that higher ER and PE are associated with increased negative cognitive appraisal of stressful situations, whereas higher EN and BR are associated with decreased the negative cognitive appraisal of stressful situations (Heszen, 2012). High-ER and high-PE individuals tend toward intensive emotional reactions and maintain states of stress, and thus, they may assess a pain-associated disease as more severe. The other three traits, BR, EN, and AC, may reduce the level of pain experienced. EN is associated with greater ability to withstand physical and social stress; BR reveals itself in an increased rate of performing different daily activities; AC serves as a direct or indirect source of stimulation (Strelau, 1993). Thus, a high level of these traits is associated with better functioning in stressful situations (Strelau, 2008).

In previous research, sensory sensitivity was not found to predict medical outcomes. Although it was found that SS tended to reduce symptom intensity in HIV+ individuals (Rzeszutek, et al., 2012), the role of SS as a predictor of medical outcomes is quite unclear. Thus, we did not create a hypothesis for this variable.

## Beliefs About Pain Control

Belief about pain control is one of the psychological factors that affects the degree of pain perception, and it is “as important to controlling of pain as control per se” (Skevington, 1990, p. 221). Although beliefs regarding internal control are more highly associated with better mental and physical health (Berglund, Lytsy, & Westerling, 2014; Milte, Luszcz, Ratcliffe, Masters, & Crotty, 2015; Reile & Leinsalu, 2013; Shojaee & French, 2014), the belief in a doctor’s control may be stronger during illness. It is assumed that the optimal health-control model includes the coexistence of an individual’s beliefs about his or her responsibility for his or her own state of health as well as beliefs about the influence of others, such as doctors. Under such conditions, the patient is aware of the availability of external support and is simultaneously motivated to be actively involved in steps to influence and control his or her health (Steuden & Okła, 2007). In a recent review, Culley, Law, Hudson, Denny, Mitchell, Baumgarten, and Raine-Fenning (2013) suggested that, among women with endometriosis, those who identify pain as abnormal, too long-lasting, and disruptive of normal functioning are more likely to seek medical help. It has been demonstrated that high chronic pain intensity is a significant factor that has a great impact on physician consultations (Andersson, Ejlertsson, Leden, & Scherstén, 1999). Intensity of pain could affect the extent to which women are able to draw on their own coping resources. Women who experience more intense pain may require consultation with a doctor and pain medication (McCrea, Wright, & Stringer, 2000). Frequency of use of health services may depend on the severity of the pain (Verhaak et al., 2000).

Some research on women with cancer suggest possible ways women with chronic painful endometriosis might respond to their health condition. Many sociodemographic factors such as age, low socioeconomic status, area of residence, and appraisal of health services may be associated with some women delaying seeking medical consultation (Kannan & Veazie, 2015; Khakbazan, Taghipour, Latifnejad Roudsari, & Mohammadi, 2014; Robinson, Christensen, Ottesen, & Krasnik, 2011). Several factors associated with help-seeking delay include psychological factors such as: lack of awareness and knowledge of the disease, disease beliefs, treatment beliefs, and emotional distress (Iskandarsyah et al., 2014). Some data showed that a high level of initial emotional response to severe illness symptoms discovery was associated with a shorter delay in seeking medical help (Meechan, Collins, & Petrie, 2003). In contrast, low fear on symptom discovery was associated with prolonged delay in women’s seeking medical aid (Li et al., 2012). Some studies have suggested that anxiety and harm avoidance may lead to delayed medical examinations in patients with painful chronic disease symptoms (Chojnacka-Szawłowska, Kościelak, Karasiewicz, Majkowicz, & Kozaka, 2013; Ristvedt & Trinkhaus, 2005). Both anxiety and harm avoidance are correlated positively with RTT traits of ER and PE, and correlated negatively with RTT traits of BR, EN, and AC (Hornowska, 2011; Strelau, 2008). Therefore, we assumed that belief in a doctor’s control may be associated with temperament trait of higher Emotional reactivity and lower Endurance. The particular role of these two traits in patients with endometriosis would arise from the fact that ER is the tendency to react intensively to emotion-inducing stimuli and EN is the ability to react adequately to intensive external stimulation and to tolerate noise, pain, temperature, or other strong stimuli (Strelau, 1993). Thus, we expected that high-ER/low-EN women, under the influence of long-term pain and its health and social consequences, would be more likely to seek medical care.

## Study Aims and Hypotheses

This study had two broad objectives: (a) to understand the relationship between temperament and beliefs about pain control and pain severity in women diagnosed with endometriosis, and (b) to explore women’s temperaments and beliefs as predictors of pain intensity ratings.

We hypothesized that: (a) emotional reactivity and perseveration would be positively correlated with the severity of pain in patients with endometriosis, while briskness, endurance, and activity would serve as buffers for pain severity, and so would correlate negatively with pain severity; (b) the intensity of patients’ pain ratings would be positively correlated with their belief that powerful others, namely, doctors, can control severe pain; and (c) the intensity of patient’s pain ratings would be less strongly correlated with their belief that they, themselves, can control severe pain. We based these expectations on the assumption that patients have greater trust in their doctors than in themselves to control severe pain.

## Materials and Methods

### Participants

The study was conducted on a group of 103 Caucasian women aged 18–44 years (*M* = 30.51; *SD* = 4.83). All were treated surgically (laparoscopy or laparotomy) in various hospitals in Poland. Medications were also used. Only women between 18 and 45 years of age (Nnoaham et al., 2011) with a laparoscopic and histopathological diagnosis of pelvic endometriosis were invited to participate. The exclusion criteria were: the presence of other diseases that induce pain; or suspected endometriosis based on an ultrasound or a history of laparoscopic surgery less than 3 months before the study. Seventy-five women (72.8 %) had postsecondary education and 28 (27.2 %) had secondary education. Sixty-eight women (66 %) were married, 35 were unmarried (34 %) and 36 had children (35 %). Fourteen women (13.6 %) lived in rural areas, 28 (27.2 %) in small towns and 61 (59.2 %) in large cities. The duration of pain symptoms in the entire sample ranged from 10 to 214 months (*M* = 83.44; *SD* = 62.1). The lapse of time from endometriosis diagnosis to psychological assessment ranged from 3 to 142 months (*M* = 45.82, *SD* = 36.96). All women continued under medical supervision and experienced pain during the period they participated in the study.

### Procedure

Participants were recruited through an announcement on the Polish Association of Endometriosis website, which included details on the research aim, target group, inclusion criteria and contact information. Before the start of the study, informed consent was collected from all women who offered to participate. We provided personal or e-mail contact information to all participants. All questionnaires were administered in a standard order. Some of the questionnaires were mailed (55 %), and the others were completed in person (45 %). The study was anonymous. Participants were not remunerated.

### Measures

#### Temperament Traits Assessment

Temperament traits were assessed with the Formal Characteristics of Behaviour-Temperament Inventory, FCB-TI (Strelau & Zawadzki, 1995). This inventory has 120 items, 20 items per scale. Respondents answered each question with yes or no (scored 1 or 0). The inventory has the following scales (Cronbach’s alpha derived from the present sample of women and the typical questionnaire items for each scale are given in parentheses).

- Briskness (BR; α = .73; When I am asked a question, I usually answer immediately; I tend to work fast, even if I have plenty of time; I often find myself hurrying, even if there is no reason to do so).
- Perseveration [PE; α = .75; My anger usually passes quickly (reverse scored); I usually remember for a long time those problems I could not solve; I often become preoccupied with one thought].
- Sensory sensitivity [SS; α = .72; I can smell even the subtlest fragrances of flowers; Whenever I stay in a new place I notice that the water tastes different; it is hard for me to make out what someone is whispering (reverse scored)].
- Emotional reactivity (ER; α = .84; I have stage fright if I have to speak in public; I get very nervous before exams or important interviews; I become more upset emotionally than most people).
- Endurance (EN; α = .83; I can concentrate on my work in spite of pain [e.g., a headache or toothache); I can continue working for a long time without a break; I feel shattered after a sleepless night (reverse scored)].
- Activity (AC; α = .80; I often engage in activities that require staying in touch with many people; I take on various professional or public functions even when they involve heavy responsibilities; I often take on very difficult tasks).

Scale scores can range from a minimum of 0 to a maximum of 20. Higher total scores in the FCB-TI scales indicated higher levels of the respective trait.

#### Measure of Beliefs About Pain Control

Beliefs about pain control were measured using the Polish version of the Beliefs about Pain Control Questionnaire (BPCQ; Skevington, 1990; Polish adaptation, Zbigniew Juczyński). This 13-item questionnaire contains three subscales: beliefs about internal or personal control of pain (IC; five items), beliefs that powerful others (doctors) can control pain (DC; four items), and beliefs that pain is controlled by chance events (CE; four items). Participants responded to each item on a 6-point Likert-type scale ranging from 1 = strongly disagree to 6 = strongly agree. Cronbach’s alpha coefficients for the Polish version, derived from the present sample of women, and the typical items for each subscale, are given in parentheses: the IC subscale (α = .80; People’s pain results from their own carelessness; I am directly responsible for my pain); the DC subscale (α = .81; I cannot get any help for my pain unless I seek medical help; Relief from pain is chiefly controlled by doctors); and the CE subscale (α = .58; Being pain-free is largely a matter of luck; No matter what I do, if I am going to be in pain I will be in pain). The IC scale total score range is 5–30, while scale scores for DC and CE may vary from a minimum of 4 to a maximum of 24. Higher total scores in the subscales indicated stronger endorsement of the respective belief.

The BPCQ questionnaire was chosen because it contains items applicable to all people, regardless of their level of education. It can also be used to test beliefs about pain control in patients who do not currently complain of pain, both in outpatient clinics and hospitals.

#### Pain Assessment

To measure the intensity of pain, the Numerical Rating Scale (NRS-11), containing 11 degrees of pain, was used. The respondent assesses the severity of pain, pointing to a number on a scale from 0 to 10, where 0 represents no pain and 10 indicates the most pain imaginable. Thus, a higher total score in this scale indicates greater pain. All women were asked to rate the average pain they experienced during their chronic disease. NRS-11 is a standard tool used in the study of chronic pain, with good evidence of clinical significance (Farrar, Pritchett, Robinson, Prakash, & Chappell, 2010; Farrar, Young, LaMoreaux, Werth, & Poole, 2001; McCaffery & Beebe, 1993; Pagé et al., 2012).

### Statistical Analysis

The statistical analysis was performed with PASW Statistics 18 (SPSS Inc., 2009). Descriptive statistics such as the means and standard deviations of the main variables are reported. Relationships among medical variables, temperament traits, and beliefs about pain control were examined with Pearson product-moment coefficients. Hierarchical multiple regression analysis was used to estimate the effect of temperament traits and beliefs about pain control as predictors of pain intensity ratings.

## Results

### Descriptive Analysis

The basic descriptive statistics for the FCB-TI scales and BPCQ and NRS-11 scales are presented in Table 1.

**Table 1:** Descriptive statistics for the FCB-TI scales and BPCQ and NRS-11 scales (*n* = 103)

Variables	Range	Mean	*SD*
FCB-TI scales			
Briskness (BR)	4–20	14.26	3.60
Perseveration (PE)	4–20	14.53	3.62
Sensory sensitivity (SS)	6–20	15.83	3.00
Emotional reactivity (ER)	0–20	13.00	4.56
Endurance (EN)	0–20	7.27	4.38
Activity (AC)	0–17	7.88	4.08
NRS-11	2–10	7.43	2.59
BPCQ scales			
Internal control (IC)	5–24	11.78	3.96
Doctors’ control (DC)	4–21	13.52	3.94
Chance events (CE)	4–21	13.17	3.81

### Relationships Between Variables

Table 2 presents Pearson correlation coefficients among the FCB-TI subscales, BPCQ subscales, and medical variables. The results presented in Table 2 show that most of the intercorrelations between the FCB-TI scales are small to moderate (an absolute value of *r* of .1 is classified as small, an absolute value of .3 is classified as medium, and of .5 is classified as large; Cohen, 1988).

**Table 2:** Pearson *r* correlations between FCB-TI scales and BPCQ scales and medical variables in the sample of women diagnosed with endometriosis

Variables	PE	SS	ER	EN	AC	IC	DC	CE	Pain intensity	Pain duration	Time diagnosis
Briskness (BR)	−.17	.23*	−.35***	.34***	.32***	−.08	−.03	.10	−.09	.01	.14
Perseveration (PE)		.14	.60***	−.33***	−.19	.03	.24**	.22**	.10	.06	−.01
Sensory sensitivity (SS)			−.01	−.05	.05	.14	−.16	−.06	−.09	−.03	.05
Emotional reactivity (ER)				−.48***	−.49***	−.05	.22*	.01	.17	.06	−.04
Endurance (EN)					.33***	.05	−.28**	−.13	−.51**	−.11	−.06
Activity (AC)						−.05	−.11	.06	−.04	.02	.07
Internal control (IC)							−.27**	−.24**	−.31**	−.21*	.00
Doctors’ control (DC)								.44***	.33**	−.01	.06
Chance events (CE)									.13	.15	.04
Pain intensity										.14	−.05
Pain duration											.56**

* p < .05; ** p < .01; *** p < .001

Regarding relationships among the six RTT traits, the correlation between PE and ER was highest. SS was correlated only with BR (small positive correlation). The positive correlations between PE and ER and positive correlations among BR, EN and AC, as well as negative correlations among PE, ER and three other traits (BR, EN and AC), were in accordance with expectations, as mentioned earlier.

Regarding relationships among the three subscales of the BPCQ, correlations between the subscales were small to moderate. The correlations between IC and DC and CE are small and negative, while the correlation between DC and CE was moderate and positive (the low Cronbach alpha for the CE subscale should be noted). Results obtained for the BPCQ scales did not appear to differ markedly from the results obtained by Skevington (1990) for the original scale.

As we can see from data in Table 2, only the endurance trait is significantly negatively correlated with pain intensity. None of the other five RTT traits were significantly related to ratings of pain intensity. Moderate correlations with pain intensity were found for internal beliefs about pain control (negative correlation) and the belief that powerful others (doctors) can control pain (positive correlation). Duration of pain was correlated only with a belief in the internal control of pain (small negative correlation). Pain intensity rating, temperament and beliefs about pain control were not correlated with duration of pain.

### Hierarchical Multiple Regression Analysis

To determine the extent to which temperament traits, and beliefs about pain control can be viewed as predictors of pain intensity in patients with endometriosis, a hierarchical multiple regression analysis was conducted. The six RTT traits were entered in Step 1 of the hierarchical multiple regression analysis, explaining 26 % of the pain intensity variance in patients with endometriosis—but as seen in Table 3, the patterning of β weights and semi-partial correlations in Step 1 indicate that that the high percentage of variance accounted for by Step 1 is due primarily to the influence of the Endurance trait. When three beliefs measured with the BPCQ were added in Step 2, the percentage of variance accounted for increased to 33 %, *F*(3, 93) = 4.07, *p* = .009.

**Table 3:** Hierarchical multiple regression analysis of temperament traits and beliefs about pain control as predictors of pain intensity in endometriosis patients (*n* = 103)

Variables	β	*R* ^2^	*ΔR* ^2^	Semi-partial correlations
Step 1		.26		
Briskness (BR)	.10			.09
Perseveration (PE)	−.02			−.02
Sensory sensitivity (SS)	−.15			−.14
Emotional reactivity (ER)	−.01			−.01
Endurance (EN)	−.61***			−.52
Activity (AC)	.13			.11
Step 2		.33	.08**	
Briskness (BR)	.05			.04
Perseveration (PE)	−.01			−.01
Sensory sensitivity (SS)	−.08			−.08
Emotional reactivity (ER)	−.07			−.05
Endurance (EN)	−.56***			−.46
Activity (AC)	.11			.10
Internal control (IC)	−.24**			−.22
Doctors control (DC)	.16			.14
Chance events (CE)	−.08			−.07

** p < 0.01; *** p < 0.001

Based on the regression coefficients, only two variables were found to be significant predictors of pain intensity assessment: endurance (β = −.56, *p* < .001) and internal control (β = −.24, *p* < .01). The results are summarized in Table 3.

Standardized beta coefficients indicate that the higher the level of endurance and internal beliefs about pain control, the lower the pain intensity ratings. The patterning of β weights and semipartial correlations in Step 2 indicates that the 33 % of variance accounted for by Step 2 is due primarily to the influence of these two predictor variables on pain intensity ratings.

## Discussion

### Main Hypotheses

The current study aimed to understand the relationships between temperament, beliefs about pain control, and pain intensity ratings in women diagnosed with endometriosis. We hypothesized that ER, PE, and doctors’ pain control were good predictors of pain intensity ratings. We also expected that three other temperament traits, BR, EN, and AC, were associated with lower levels of pain ratings by women with endometriosis.

Contrary to expectations, ER and PE were not significantly related to pain intensity ratings. However, the perception of pain, according to our hypothesis, was negatively correlated with endurance, another one of the temperament traits. Endurance was the only temperament trait significantly (negatively) correlated with pain intensity ratings. The ability to respond adequately, even in conditions of extreme distress, which is the essence of EN, proved to be an important buffer for the intensity of pain experienced. Probably, greater resistance to pain and behaviors resulting from endurance as a temperamental trait allow for better control of pain. Consequently, better control of pain can lead to a low subjective rating of pain intensity. An analysis of the correlations between the scales that measure the temperament traits indicated that BR, EN, and AC may serve similar functions in the structure of temperament, but that briskness and activity were not significant for the experienced pain intensity rating. Perhaps these two traits have more influence when individuals cope with distress that results from general life changes instead of specific circumstances, such as an illness. In the case of pain, we have to deal with the intense, ever-persistent stimulation, and effective pain control requires resilience and a capacity for long-term operation under adverse conditions (and therefore a high level of endurance).

The finding that ER and PE are unrelated to pain intensity ratings may indicates possibly different roles for EN, ER, and PE in the presence of chronic pain. Resistance to such distractors as pain is the essence of EN (Strelau, 2008). However, the significance of ER and PE may be more related to the assessment of the consequences of long-term pain and may lead to emotional reactions, such as pain-related fear or pain-related anxiety and negative mood, which often accompany chronic pain (Ramirez-Maestre & Esteve, 2014). Consequently, the emotional response to pain may be associated with the belief that doctors can control pain, as suggested by the results of our study, and may lead to the involvement of the patient in treatment. We did not examine this aspect of the role of ER and PE, although a relationship between these traits and anxiety was demonstrated by Strelau and Zawadzki (2011).

Our findings suggest that beliefs about pain control also influence the intensity of the pain experienced. Pain intensity ratings appear to depend on a patient’s beliefs about her own ability to control pain, that is, internal pain control, as well as her beliefs about doctors’ ability to control pain. However, the direction of these relationships appears to be different. Women who perceive themselves as having greater control of their pain tend to rate pain severity lower. Patients who rated pain as stronger had greater trust in doctors’ ability to control pain. An unexpected finding was that longer durations of pain were associated with decreased belief in one’s own ability to control pain. This result merits further exploration in future studies.

Our results are in line with the hypothesis that the more patients believe they can control their pain (i.e., the greater is the perceived internal locus of pain control), the less the perceived severity of their pain will be (Allison, 1991). However, the more patients believe that doctors can control patients’ pain (i.e., the greater is the perceived external locus of pain control), the greater will be the perceived intensity of patients’ pain, and which, in turn, may adversely affect their quality of life and medical status (Steuden & Okła, 2007).

Our findings are correlational and therefore do not indicate the direction of causality. Though we have emphasized that perceived locus of control can affect the perceived pain intensity, it is also possible, even likely, that the perceived intensity of pain can affect the perceived locus of control. In other words, patients who experience greater pain may perceive themselves as having less control over their pain and may perceive doctors as having more control over the pain. Moreover, the relationship between pain and perceived locus of control could be bidirectional, with perceived control variables influencing pain severity and pain severity influencing perceived control. Perhaps more intense pain reduces patients’ belief that they are able to control their response to pain and, instead, increases their belief that doctors (powerful others) are better able to control pain. If so, our results may be in line those authors who suggest that the amount of pain experienced may determine the method of pain relief that patients use, e.g., using either their own coping resources or seeking assistance from doctors (Andersson et al., 1999; McCrae et al., 2000).

In the present study, the rating of pain as a symptom of endometriosis was significantly predicted by two psychological factors. This is indicated by the results of the hierarchical multiple regression analysis. The endurance trait and beliefs about internal pain control accounted for 33 % of the variance in pain intensity ratings in women with endometriosis. A key role is played by the factor of individual human endurance in conditions of strong and/or long-term stimulation. Internal beliefs about pain control seem to be independent of temperament and are probably determined by the other variables, such as cognitive or situational factors, like chronically pain illness. As Skevington (1990) suggested, among chronic pain patients, very low scores on the IC scale may indicate that severe pain may undermine patients’ beliefs in their personal ability to control pain. Our results suggested, therefore, that beliefs about internal pain control and endurance, one of the temperament traits, are important for the perception of pain by patients with endometriosis.

### Methodological Limitations

Several limitations of our study must be noted. First, the sample under study was small and self-selected and therefore may not be representative of the entire population of women with endometriosis in Poland. Second, there are many important social and interpersonal variables that can influence ratings of pain severity whose effects we did not take into account. For example, social support (Chao, Abercrombie, & Duncan, 2012) can powerfully influence quality of life and pain-related stress. Third, there is a range of additional medical variables whose role we did not consider such as severity of endometriosis, or the influence of pharmacological management of pain, factors that could strongly influence patients’ perception of physicians’ ability to control pain.

In addition, we propose extending the research to additional variables. It might be interesting to determine the role of temperament and social support in coping with endometriosis, especially if endometriosis is not understood by society to be an important source of pain (Barnack & Chrisler, 2007). The role of temperament in the coping process may be particularly interesting because of the relatively high anxiety levels observed in women with endometriosis. It has been demonstrated that this group of women is often quite easily stressed and anxious (Low et al., 1993; Sepulcri & Amaral, 2009), especially when endometriosis is associated with migraines (Tietjen et al., 2007). Moreover, it is suggested that menstrual irregularities as symptoms of endometriosis can be confused with infertility and make women vulnerable to stigma (Seear, 2009).

### Possible Clinical Implications

Regardless of the methodological limitations of our study, however, we believe that the results obtained may be useful in understanding how some psychological factors influence women’s reports of pain in endometriosis. Beliefs about internal pain control may prove to be a useful target in psychological interventions to improve personal control over pain and to extend control over the disease and its course.
